# Does heat tolerance vary with rates of oxygen production in photosymbiotic cnidarians?

**DOI:** 10.1242/jeb.251544

**Published:** 2026-02-12

**Authors:** Elise M. J. Laetz, Wilco C. E. P. Verberk

**Affiliations:** ^1^ Groningen Institute for Evolutionary Life Sciences, University of Groningen, Nijenborgh 7, 9747 AG, Groningen, The Netherlands; ^2^ Radboud University Nijmegen, Heyendaalseweg 135, 6525 AJ Nijmegen, The Netherlands

**Keywords:** Photosymbiosis, Anthozoa, Hydrozoa, Scyphozoa, OCLTT, CT_max_

## Abstract

Oxygen acquisition and delivery to tissues is believed to be a key factor in heat tolerance, but testing this link has been challenging owing to methodological limitations to separate processes related to oxygen acquisition and oxygen delivery. In this study, we altered tissue oxygenation by manipulating light intensity using cnidarians that host endosymbiotic algae as model species. We first verified that light intensity determines net photosynthetic rates, showing that all species produced oxygen at the highest light intensity, and that chemically inhibiting photosynthesis successfully reduced oxygen production. We then tested the prediction that heat tolerance would be higher at higher light intensities and lower in specimens that no longer have internal oxygen production due to photosynthesis (chemical inhibition). Overall, photosynthetic specimens had a higher heat tolerance than inhibited specimens and increased light intensity improved heat tolerance for two of the three species we examined. Because inhibited specimens had lower heat tolerances, we conclude that oxygen dynamics are involved in shaping heat tolerance. Interestingly, light intensity also affected oxygen uptake and heat tolerance in some of the chemically inhibited specimens, indicating that either we did not achieve complete inhibition of photosynthesis or that light modulates aspects of cnidarian metabolism that are related to thermal tolerance, but which may extend beyond oxygen dynamics and the photosynthesis occurring in their algae.

## Teaser

**Summary:** Manipulation of oxygen production in photosynthetic cnidarians demonstrates an empirical link between thermal tolerance and oxygen availability, although oxygen was not the sole determinant of thermal tolerance.

## INTRODUCTION

Assessing the vulnerability of organisms to global warming frequently employs experimental measurements of their heat tolerance. With thermal isoclines being major correlates of patterns in species distribution, it is perhaps not too surprising that patterns in heat tolerance are related to geographic distribution ranges of species and their habitat use (Deutsch et al., 2008; Leiva et al., 2019; Pinsky et al., 2019). In aquatic (i.e. marine and freshwater) ectotherms, such linkages between heat tolerance and geographic distribution appear to be stronger than for terrestrial ectotherms (Sunday et al., 2012).

One mechanism that has long been hypothesized to explain the decrease in performance and eventual mortality in ectotherms exposed to stressfully high temperatures is a shortage of oxygen (Winterstein, 1905). Elevated temperatures lead to exponential increases in biological rates, with concomitant increases in metabolic demand for energy and hence oxygen (Clarke and Fraser, 2004). According to the oxygen- and capacity-limited thermal tolerance (OCLTT) hypothesis, beyond some upper temperature threshold, animals have insufficient capacity for extracting and transporting oxygen such that oxygen delivery can no longer keep up with elevated tissue demand for oxygen (Pörtner, 2001). Although the hypothesis was originally proposed to be a universal explanation for heat tolerance limits under normoxia, experimental support for this hypothesis is mixed and appears to be stronger, but not universal, for aquatic ectotherms than for terrestrial ectotherms (Verberk et al., 2016). Note that although less oxygen dissolves in warmer water, maximum rates of diffusion can still increase as a result of an increase in oxygen diffusivity (Verberk et al., 2011). However, extracting oxygen from water in general is much more challenging than from air, which likely contributes to the greater susceptibility to oxygen limitation in water breathers (Leiva et al., 2019).

In addition to measurements of aerobic and anaerobic metabolism to characterize thermal limits, manipulative approaches to testing the oxygen limitation hypothesis comprise altering either ambient oxygen availability or the capacity of organisms to extract and transport oxygen (Brijs et al., 2015; Verberk et al., 2013). Although experiments often show strong reductions in heat tolerance when measured in water in which oxygen levels are reduced below 100% saturation, improvements in heat tolerance when measured in hyperoxic water are typically much less pronounced, or absent (Verberk et al., 2016). Experiments with hyperoxia alleviate potential oxygen deficiencies and are therefore seen as a stronger test of the oxygen limitation hypothesis. However, hyperoxia itself can be stressful and alleviating oxygen limitation can simply result in a process other than oxygen delivery to become limiting or detrimental (e.g. protein denaturation or altered membrane permeability) (Verberk et al., 2013). If the temperatures at which both processes fail are similar, no substantial improvement in heat tolerance is expected, but oxygen may still be limiting under normoxia. Note also that if organisms only infrequently experience hyperoxic conditions, they are unlikely to have evolved the supply capacity to exploit periods of hyperoxia (Seibel and Deutsch, 2020); however, this is unlikely in species inhabiting tropical waters because of large, diel fluctuations in oxygen bioavailability due to photosynthesis and respiration cycles. In a similar vein, a few studies have manipulated the capacity of an organism for oxygen transport and then tested whether this affected their heat tolerance. These found subtle changes (e.g. Wang et al., 2014), but because the effects were small, it has remained difficult to falsify the hypothesis.

**Fig. 1.**
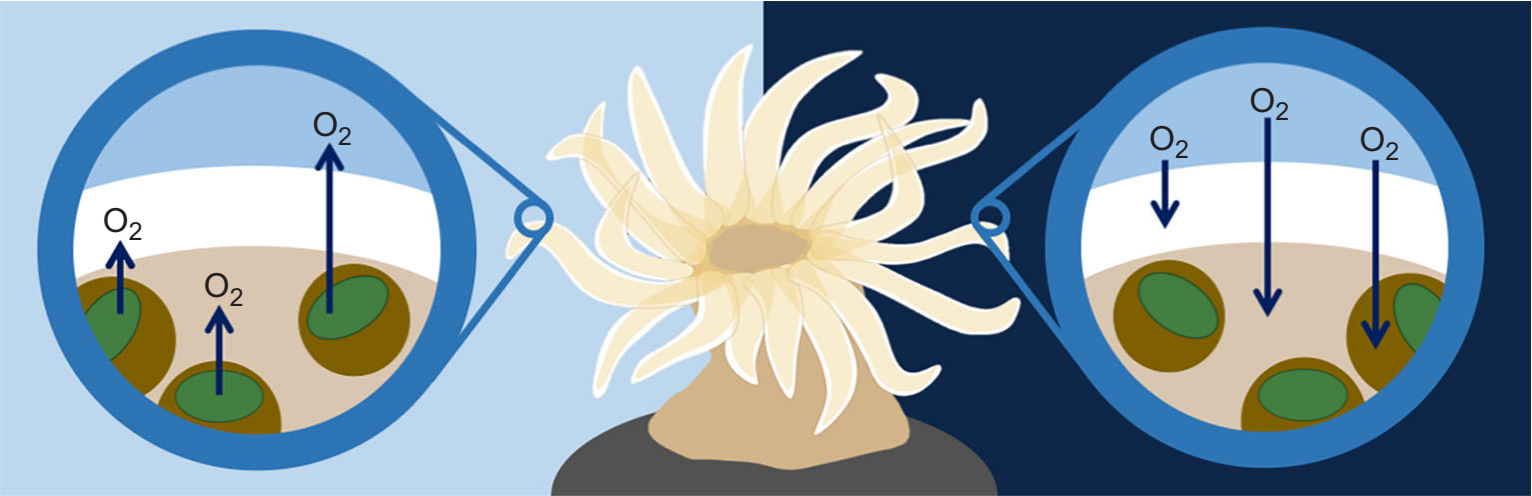
**Schematic view of oxygen dynamics in a photosymbiotic sea anemone.** During the day (left), algae (brown circles) produce O_2_ in their chloroplasts (green) via photosynthesis. This O_2_ surpasses the amount needed for algal respiration, so the surplus O_2_ diffuses (dark blue arrows) into the sea anemone’s gastroderm cells, where the symbiotic algae are housed (beige tissue layer), and ectoderm cells (white tissue layer), where it is used in aerobic respiration. Excess O_2_ diffuses into the surrounding seawater (light blue). At night, both the anemone and the algae take up O_2_ from the surrounding seawater, releasing CO_2_ during respiration, which diffuses into the surrounding seawater. Although their anatomy differs, similar oxygen dynamics occur in photosymbiotic hydrozoans and scyphozoans.

To empirically test the OCLTT hypothesis and circumvent these experimental difficulties by noninvasively altering tissue oxygenation without manipulating the oxygen levels in the water, or an organism's capacity to transport oxygen, we examined three different photosynthetic cnidarian species spanning diverse clades (Anthozoa, Scyphozoa and Hydrozoa) under different light intensities. The gastrodermal tissue of these cnidarians is filled with single-celled dinoflagellates that can photosynthesize to produce sugars and oxygen in a light-dependent manner (Fig. 1). We hypothesized that if oxygen is limiting under normoxia, these species should exhibit improved heat tolerance with increasing light intensity as more and more oxygen is generated internally as a byproduct of photosynthesis (providing the amount of light does not exceed the amount needed to induce photoinhibition and photodamage). In previous work on a sea slug that retains functional chloroplasts from the algae on which it feeds, we observed improved heat tolerance under high light intensity (Laetz et al., 2024). In this proof of principle investigation, we expand on this work by studying three different species, and as an additional test, we chemically inhibited photosynthesis to investigate whether photosynthesis, not light, determines heat tolerance.

## MATERIALS AND METHODS

**Fig. 2.**
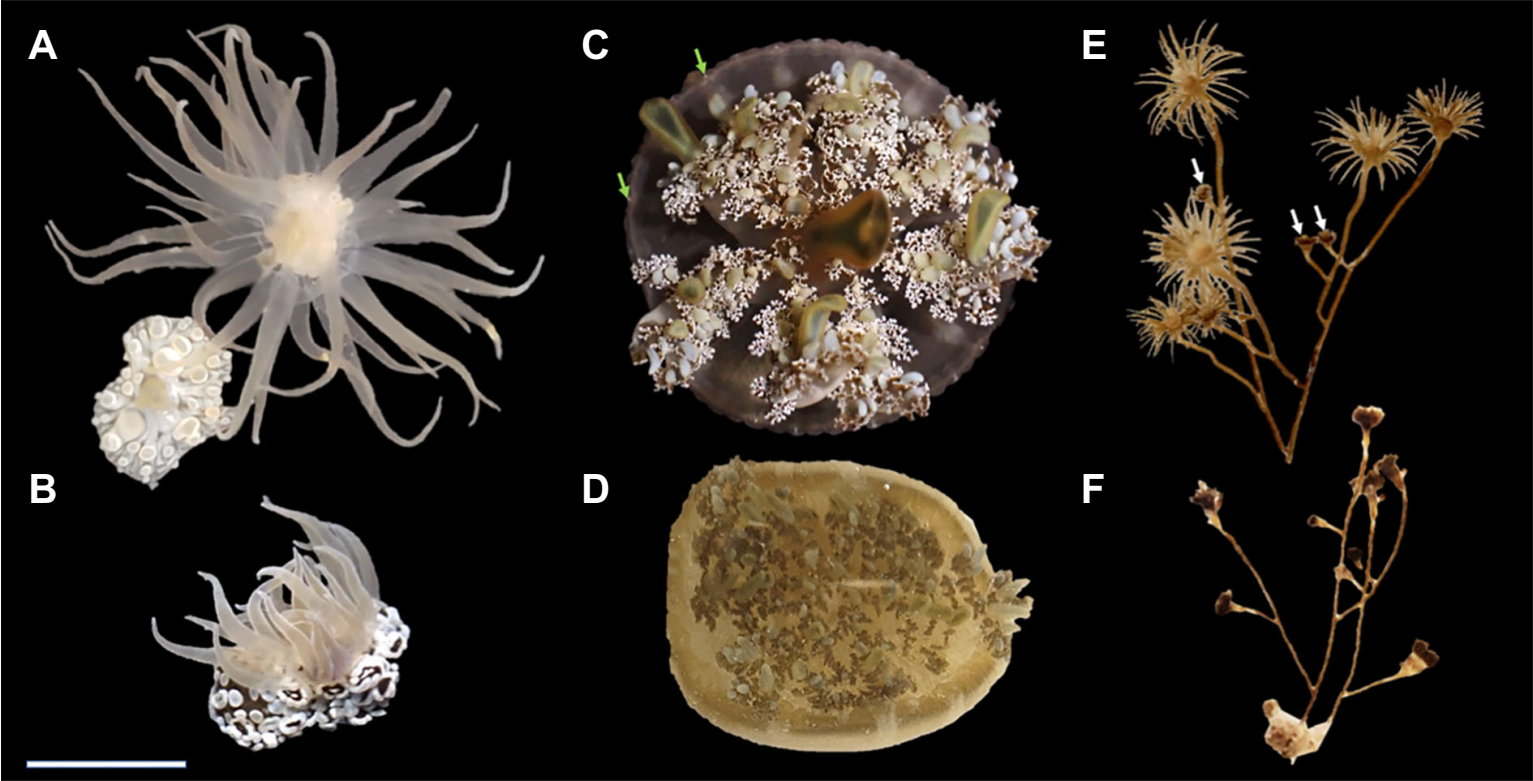
**The species used in this study and the behaviors used to define critical thermal limits CT1 and CT2.** (A) *Bunodeopsis antilliensis* (oral view) fully extended and completed contracted (lower left). Specimens were observed in both of these states in the field and the holding tank, but they always had tentacles completely extended after transfer to the jars used in the critical thermal limit trials. (B) The partial contraction of all tentacles in one sudden movement (CT1) and the point at which the pedal disc detached from the respirometry chamber wall or bottom (CT2). (C) *Cassiopea* cf. *xamachana* oral view during bell relaxation and (D) during a bell pulse. The green arrows indicate rhopalia (light sensing organs). (E) A *Myrionema hargitti* specimen that simultaneously retracted three small polyps (CT1, white arrows) and (F) a specimen with all of its polyps retracted (CT2). Scale bar: (A) ∼2 mm; (B) ∼4 mm; (C,D) ∼15 mm; and (E,F) ∼10 mm.

Three different cnidarian species (*n*=54 specimens per species) were collected from the same mangrove habitat in Spaanse Water Bay (12.076302, −68.860797), a saline (35 PSU) bay on Curaçao in April 2022 (Fig. 2). The anthozoan *Bunodeopsis antilliensis* Duerden 1897 was found on *Thalassia testudinum* and *Halophila stipulacea* seagrass at a depth of 0.5–1.5 m. The hydrozoan *Myrionema hargitti* (Congdon 1906) was attached to mangrove roots at 0–1 m deep, and the scyphozoan *Cassiopea* cf. *xamachana* Bigelow 1892 was found amongst the seagrass rhizoid and on bare patches of silt at 1–2 m depth. The light intensity at the collection site was measured between 12:00 and 12:33 h on the first day specimens were collected using a SQ-500-SS Full-spectrum Quantum PAR (photosynthetically active radiation) sensor with a 30 cm cable and a microCache Bluetooth micro-logger (Apogee Instruments) as described in Burgués Palau et al. (2024). Five measurements were taken at 0, 2 and 4 m depth and the average light intensity at 2 m deep was used to define the high light treatment described below. The water temperature was also recorded at 26°C using the thermometer belonging to a Fibox 4 Trace (detailed below; Presens, Germany). All specimens were transported in individual jars containing approximately two-thirds seawater and one-third air, to the laboratory at the Caribbean Research and Management of Biodiversity Institute (CARMABI) for experimentation. All applicable international, national and/or institutional guidelines for the care and use of animals were followed.

**Fig. 3.**
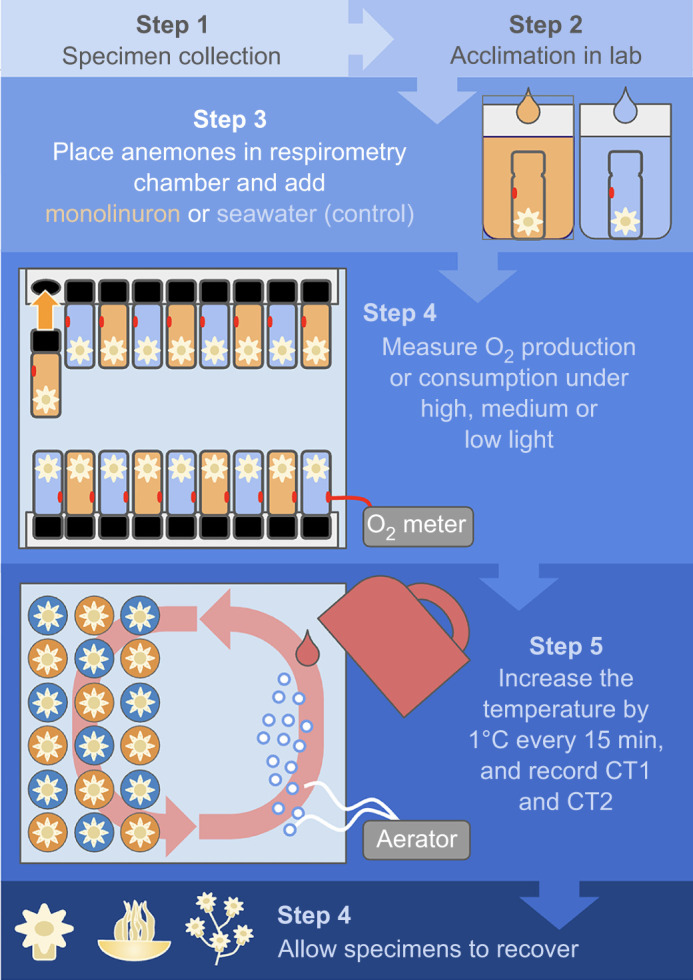
**Experimental overview.** Each specimen used in this study was handled according to this pipeline. Specimens were collected, transported to the lab and allowed to acclimate to lab conditions. They were then treated with monolinuron or seawater as a control, and placed in open respirometry chambers to adjust before the chambers were sealed and each specimen's oxygen production or consumption was recorded. They were then placed in individual jars for the critical thermal limit experiment. Once their critical thermal limits (CT1 and CT2) were reached, jars were removed from the warm water bath and placed in a cool water bath to cool down, allowing specimens to recover.

In the lab, specimens were housed in 20 liter plastic tanks (separated by species) containing aerated seawater maintained at 26°C to match the water temperature in the field when they were collected. They were provided with a light intensity of 1600 µmol m^−2^ s^−1^ (30 W full spectrum LED, OSRAM, Germany), matching the light intensity measurements taken at 2 m depth in the field at midday on the first day samples were collected and used in these experiments. Specimens were allowed to adjust to laboratory conditions for at least 3 h before experimentation began and experiments were conducted at the same time every day, starting at 17:00 h and ending around 19:00 h, when the photoperiod naturally ends. This allowed sufficient time to adjust to laboratory conditions following collection, and for maximum photosynthetic energy production at the end of the photoperiod, without extending their light exposure beyond what they receive in nature. We measured 18 specimens per day, nine in monolinuron and nine that were untreated, such that one light treatment was completed each day; an overview of the experimental process and setup is presented in Fig. 3. Half of these specimens remained capable of photosynthesis during experimentation (i.e. untreated). The other half of the specimens was treated with a 2 μg ml^−1^ solution of monolinuron (Algol, JBL, Germany) in seawater for 2 h before they were measured (Christa et al., 2014). Monolinuron selectively inhibits photosystem II activity by blocking the electron transport chain (Arrhenius et al., 2004), which also blocks O_2_ formation. Exposure to 2 µg ml^−1^ has demonstrated effectiveness in chloroplasts, even when they are incorporated in animal tissues (Christa et al., 2014; Laetz et al., 2017). Because it was unclear whether this concentration would work in diverse cnidarians as previous experiments using this concentration (2 µg ml^−1^) examined sea slugs or far higher concentrations of a similar aryl urea based herbicide (diuron) in corals (e.g. 10–100 µg l^−1^) with documented negative effects (Råberg et al., 2003), our first research question examined whether exposure to 2 µg l^−1^ monolinuron markedly increased their need to take up O_2_ from the surrounding seawater, indicating that we had at least partially achieved photosynthetic inhibition. Because this difference was observed, we continued with the 2 µg l^−1^ concentration for all of the light treatments to avoid the negative effects observed at higher concentrations in corals. We hereafter refer to untreated, control specimens as ‘photosynthetic’ and monolinuron-treated specimens as ‘photosynthetically inhibited’.

### Oxygen production/consumption

Nine specimens from each species were randomly selected for each treatment according to a 3×2 experimental design (three light conditions×monolinuron-treated or not monolinuron-treated; Table S1). Light intensity was manipulated by placing 30 W LED panels (OSRAM) at different heights above the specimens, resulting in the provisioning of 100, 700 and 1600 µmol m^−2^ s^−1^ of light matching natural conditions. Specimens were first examined for oxygen production/consumption (respirometry trials) and then placed in the critical thermal limit trials described in the next section.

To measure the metabolic rate of each specimen, it was placed in a respiratory chamber (glass jar) containing aerated seawater (with or without monolinuron) and a PSt3 optical sensor (Presens) and given 30 min to adjust to the chamber (preliminary tests showed that animals needed to ∼20 min before their metabolism fell to unstressed levels after being relocated). The largest species had an average diameter of ∼20 mm when contracted, thus *C.* cf. *xamachana* were measured in chambers that held 22 ml of seawater, whereas *B. antilliensis* and *M. hargitti* (polyp diameter of ∼8 and ∼2 mm, respectively) were measured in smaller chambers holding only 2 ml of water. These chambers were placed in foam holders attached to the side of the water bath, which maintained a constant temperature of 26°C and ensured that every jar was at the same depth receiving the same light intensity. Placing the chambers sideways also ensured that the specimen received full light (i.e. the light was not blocked by the cap) and that the sensor did not receive full light, because it was positioned perpendicular to the light source. A Fibox 4 Trace (Presens) was used to record the oxygen saturation every 6 min for 1 h. Before experimentation, the Fibox was calibrated using two-point calibration (100% O_2_-saturated seawater and 0% oxygen:nitrogen gas). Oxygen saturation was converted to concentration following the protocol outlined in Laetz et al. (2024) and detailed in the Supplementary Materials and Methods. Respirometry chambers filled only with seawater from the acclimation tank (the same seawater that was used in the specimen-containing chambers) were also measured to determine how much oxygen was consumed or produced by microbes living in the seawater. These values were subtracted from the net change in oxygen saturation so the values we present in the results refer only to the oxygen produced/consumed by the cnidarian. Typically, water is stirred during the period when the respirometry chambers are closed in order to prevent pockets of low or high dissolved oxygen water accumulating owing to boundary layer effects. However, our study species were fragile and as a result we decided against stirring the water during respiration trials to prevent mechanical stress. Lack of stirring may have introduced noise into our measurements. To check whether this was problematic, we paid special attention to the oxygen traces for each specimen. These showed a consistent, mostly linear change in oxygen saturation within each chamber with good statistical fits (i.e. the mean *R*^2^ was 0.88), indicating that lack of stirring did not represent a major methodological issue in our study.

### Critical thermal limits

Following the respiration trials, specimens were transferred from the respirometry chambers into individual jars at one end of a large plastic container and given 30 min to adjust to their new container. These jars were maintained at 26°C during this acclimation period. The water temperature was then increased by 1°C every 15 min adding small amounts of boiling water to the other end of the container. Vigorous aeration ensured rapid water mixing. Specimens were allowed to recover for an hour after experimentation to ensure the trial was not lethal and all of our specimens survived these trials.

Two behavioral changes were used to denote each species' critical thermal limits (CT1 and CT2). In *B. antilliensis*, unstressed specimens were attached to the substrate via their pedal discs and they often had their tentacles extended into the water column (Fig. 2A). CT1 was defined as the temperature at which each anemone partially and suddenly retracted all of its tentacles at once, a stress response they also demonstrated when being moved from their substrate (Fig. 2B). CT2 was defined when *B. antilliensis* detached from the substrate (Fig. 2B). In *C.* cf. *xamachana*, unstressed specimens pulsed their bells 40–60 times per minute (Fig. 2C). When thermally stressed, they reduce this rate to <10 times per minute (CT1). After additional warming, jellyfish no longer reacted to a soft jet of water from a pipette by pulsing their bells rapidly, indicating they were no longer able to try to flee the stressor (CT2; Fig. 2D). In *M. hargitti*, unstressed specimens were observed with their polyps fanned out in the water. CT1 was defined as the temperature at which each specimen retracted the first polyp(s) (Fig. 2E) and CT2 was defined as the point at which it retracted all of its polyps (Fig. 2F). These behaviors were determined during preliminary observations of both unstressed animals and those subjected to increasing temperatures. Additional behaviors that were examined in *C.* cf. *xamachana* included long pauses in bell pulses, uniformity of bell pulse (i.e. whether all parts of the bell contracted evenly) and pulse speed. However, these behaviors varied greatly among unstressed specimens and were excluded as indicators of heat stress. Similarly, *B. antilliensis* and *M. hargitti* retracted individual tentacles and *B. antilliensis* discharged acontia when perturbed, but these behaviors did not change when subjected to increasing temperatures and were therefore excluded as well.

### Data analysis

Both oxygen production/consumption and the critical thermal limits were analyzed in RStudio (https://posit.co/download/rstudio-desktop/) based on R version 4.2.0 (https://www.r-project.org/) using linear models. Best fit models were identified using Akaike information criterion values. The model that best fit our O_2_ data explained 85% of the variation and included the fixed effects, light intensity, monolinuron, species and their interactions. Because CT1 and CT2 were positively correlated (Pearson *r*=0.694, *t*_1,159_=12.17, *P*<0.0001), they were pooled (both analyzed simultaneously) and the resulting model explained 84% of the variation. In both analyses, light intensity was log transformed to allow curvilinear relationships to be fitted. As we found significant three-way interactions between light, species and monolinuron for both sets of data (metabolic rates and critical thermal limits), we proceeded to fit simplified models to evaluate their effects for each species separately. We did this by subsetting the data by species and running the same model excluding species and its interactions. Significance levels were then based on a Type III ANOVA, unless the interaction between light and monolinuron was not significant, in which case we used a Type II ANOVA (detailed in Table 1, Tables S2 and S3 and the code provided in DataverseNL: https://doi.org/10.34894/UPYAUJ). The packages ggplot2 (Wickham, 2016), dplyr (https://CRAN.R-project.org/package=dplyr), broom (https://CRAN.R-project.org/package=broom) and visreg (Breheny and Burchett, 2017) were used for data handling and visualization.

**Table 1.** ANOVA results for *Bunodeopsis antilliensis*, *Cassiopea* cf. *xamachana* and *Myrionema hargitti*

		Light intensity		Monolinuron		Monolinuron×Light intensity		Type of ANOVA
Response variable	Species	*F*	*P*	*F*	*P*	*F*	*P*	
Metabolic rate	*B. antilliensis*	28.95	**<0.0001**	105.71	**<0.0001**	1.56	0.22	II
	*C.* cf. *xamachana*	24.20	**<0.0001**	7.78	**0.0075**	35.28	**<0.0001**	III
	*M. hargitti*	39.20	**<0.0001**	1.92	0.172518	23.63	**<0.0001**	III
Critical thermal limit	*B. antilliensis*	14.51	**0.00024**	2.04	0.15593	6.07	**0.015**	III
	*C.* cf. *xamachana*	137.48	**<0.0001**	34.56	**<0.0001**	0.51	0.48	II
	*M. hargitti*	6.42	**0.013**	44.19	**<0.0001**	0.014	0.91	II

*P*-values in bold are <0.05 and thus considered significant.

## RESULTS

### Light intensity

Light intensity at the collection site decreased with depth from 1986.58±154.72 µmol m^−2^ s^−1^ (mean±s.d.) at the water's surface to 1624.45±207.74 µmol m^−2^ s^−1^ at 2 m depth and 1277.62±149.70 µmol m^−2^ s^−1^ at 4 m depth (Table S4).

### Oxygen production/consumption

**Fig. 4.**
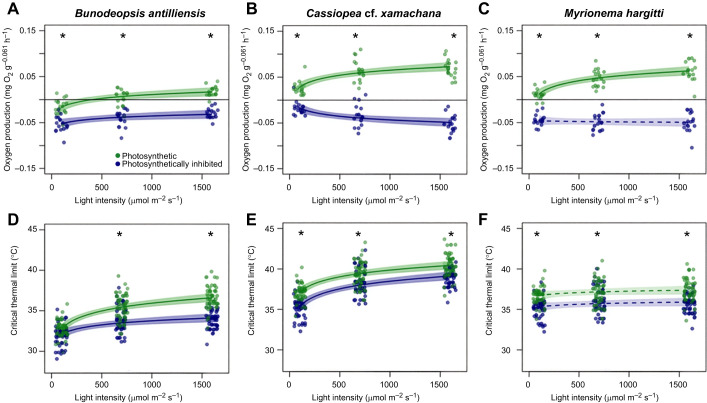
**Effects of light intensity on oxygen production/consumption and heat tolerance in the three study species.** (A–C) Oxygen production/consumption and (D–F) heat tolerance (°C) in relation to light intensity for *Bunodeopsis antilliensis* (A,D), *Cassiopea* cf. *xamachana* (B,E) and *Myrionema hargitti* (C,F). In each panel, we distinguish between photosynthetic specimens (green points and shading), and those incubated with monolinuron to inhibit photosynthesis (blue points and shading). For A–C, positive values indicate (net) oxygen production, and negative values indicate (net) oxygen consumption. In D–F, CT1 and CT2 were pooled because they were highly correlated. All panels depict partial residual plots, which illustrate the relationship between the response variable and an independent variable while accounting for the effects of other independent variables in the model (Tables S3 and S4). The lines are regression lines and the shaded bands are 95% confidence intervals. Dashed lines and lighter shaded bands indicate that no significant relationship exists between the points connected by that line. Asterisks in the upper part of the plot indicate treatments where monolinuron treated samples differed significantly from non-inhibted (photosynthetic) specimens (*P*<0.05). All treatments except for *B. antilliensis* at 100 µmol m^−2^ s^−1^ displayed significantly lower oxygen production and thermal limits when photosynthesis was inhibited.

All three cnidarian species were able to oxygenate the surrounding seawater when photosynthesis was uninhibited and animals were provided with high light intensities (700 or 1600 µmol m^−2^ s^−1^), as evidenced by positive O_2_ production values. Under low light (50 µmol m^−2^ s^−1^), *B. antilliensis* consumed more O_2_ than was produced via photosynthesis, whereas *C.* cf*. xamachana* and *M. hargitti* were net O_2_ producers even at the lowest light intensity*.* The amount of O_2_ that was produced by each species increased at higher light intensities for all three species (*t*>4.13, *P*<0.001; Fig. 4A–C). Monolinuron drastically reduced rates of photosynthesis, resulting in a net uptake of O_2_ from the surrounding seawater (i.e. negative O_2_ production values; Fig. 4A–C; Table S2). Rates of O_2_ consumption in photosynthetically inhibited animals were either weakly positively related to light intensity (*B. antilliensis*; Fig. 4A), did not respond to light intensity (*M. hargitti*; Fig. 4C) or were weakly negatively related to light intensity (*C. xamachana*; Fig. 4B).

### Thermal tolerance

Photosynthetic *B. antilliensis* and *C.* cf. *xamachana* showed improved heat tolerance with increasing light intensity (*t*>7.50, *P*<0.0001), whereas *M. hargitti* showed a non-significant relationship (*t*=1.5, *P*=0.133). Photosynthetic specimens also displayed higher heat tolerance when compared with photosynthetically inhibited specimens, for each species under medium and high light (Fig. 4D–F). Surprisingly, light intensity also affected heat tolerance in photosynthetically inhibited specimens of *B. antilliensis* and *C.* cf. *xamachana*. In *B. antilliensis*, a significant interaction between monolinuron and light intensity (*F*=6.07, *P*=0.015) indicates that light intensity had a much smaller effect on heat tolerance in photosynthetically inhibited specimens compared with uninhibited specimens.

## DISCUSSION

### Light intensity and oxygen saturation in the field

Because specimens were collected at 1–2 m depth, the highest light intensity treatment in this study (1600 µmol m^−2^ s^−1^) matched these conditions and was not considered an environmental stressor for these species, despite the fact that it is higher than the light intensities used in most photophysiological studies. This reinforces the notion that most photophysiological studies chronically underestimate the light intensities found in nature (Burgués Palau et al., 2024; McLachlan et al., 2020). Furthermore, if exposure to this intensity was a stressor, we would expect a decrease in O_2_ production rates at higher light intensities because of photoinhibition (Booth et al., 2023) (Fig. 4A,C,E); however, this was not observed.

### Oxygen production/consumption

At 700 and 1600 µmol m^−2^ s^−1^, all of the specimens that were allowed to photosynthesize produced more O_2_ than they consumed, oxygenating the surrounding seawater. This indicates that photosynthesis generated O_2_, which difused out of the algae into the animal host and then into the surrounding seawater, causing its net O_2_ saturation to increase. At 100 µmol m^−2^ s^−1^, most specimens of *C.* cf. *xamachana* and *M. hargitti* still oxygenated the surrounding seawater, but most *B. antilliensis* did not, meaning they took up O_2_ from the surrounding seawater.

When photosynthetically inhibited, all species took up O_2_ from the seawater, as evidenced by the decreasing oxygen saturation values we measured in the surrounding seawater. Although we cannot assess whether full photosynthetic inhibition was achieved with our short and relatively low dose of monolinuron, the discrepancy between oxygen-producing (photosynthetic) specimens and oxygen-consuming (photosynthetically inhibited) specimens in all three species indicates that photosynthesis no longer met their aerobic demands and they made up for this shortfall by taking up O_2_ from their environments. Partial inhibition may explain why light still modulated the rates of O_2_ consumption in photosynthetically inhibited specimens, although there could be other explanations as to why these animals change their metabolisms under varying light conditions. Regardless of whether inhibition was complete, the differences in oxygen dynamics (production versus consumption) and heat tolerance between photosynthetically inhibited and photosynthetic specimens indicate that the dosage used in this study sufficiently manipulated internal O_2_ levels in their tissues, which were higher in photosynthetic individuals. Off-target effects of monolinuron on host mitochondrial function could potentially influence our results. For example, Råberg et al. (2003) reported decreases in coral host respiration following exposure to diuron, an aryl urea herbicide similar to monolinuron. However, given the relatively low dosage and especially the short duration of 2 h we used in comparison (the Råberg study exposed animals for multiple days and concentrations of 10–100 µg l^−1^), we do not expect off-target effects of monolinuron.

In our study, we decided not to stir the water inside each respirometry chamber to prevent mechanical stress of stirring, which could harm soft-bodied cnidarians. However, if the water is not thoroughly mixed, this could lead to oxygen gradients forming in the chamber, which could have introduced noise in our measurements because the optode sensors we used measured the oxygen conditions at the inner surface of the glass chambers. Despite this, we have little evidence that such noise was present in our observations. *Cassiopea* cf. *xamachana* consistently pulsed their bells during this experiment, which ensured that their seawater was mixed. Oxygen gradients could have formed in the chambers housing *M. hargitti* and *B. antilliensis* as they do not move as much; however, we observed oxygen generation in all three species, indicating that boundary effect noise, if present, did not mask the clear effects of light and photosynthetic inhibition that we observed. Furthermore, if relevant oxygen gradients were present, our reported values underestimate the actual rates of oxygen production, because oxygen produced via photosynthesis would create an oxygen gradient which is higher at the boundary layer enveloping the animal and lower at the edge of the respirometry chamber where our optode was situated.

### Heat tolerance

In general, we observed that photosynthetic specimens displayed a greater ability to withstand heat stress than photosynthetically inhibited specimens (Fig. 4D–F) and their heat tolerance exceeded the maximum temperatures that they are exposed to in the field, which ranges from 27.0 to 31.4°C (Nagelkerken et al., 2000). We expected that amongst photosynthetically inhibited specimens, heat tolerance would be similar to that observed at the lowest light intensity for uninhibited specimens and heat tolerance would no longer improve with increasing light intensity. *Bunodeopsis antilliensis* fits this expectation best: at the lowest light intensity, heat tolerance was comparable between photosynthetically inhibited and uninhibited specimens and heat tolerance only weakly improved with increasing light intensity in photosynthetically inhibited specimens. Both other species showed a lower heat tolerance in photosynthetically inhibited specimens at high light intensity as was predicted, but heat tolerance was also reduced at low light intensity, which was not predicted. This could indicate that photosynthesis was not completely inhibited and even reduced rates of photosynthesis may have elevated internal oxygen levels of the tissue layer holding the symbionts, especially if our measurement underestimated the actual oxygenation. This could explain how light affected heat tolerance in photosynthetically inhibited specimens of *B. antilliensis*, because light also reduced rates of oxygen uptake in photosynthetically inhibited specimens in this species. Taken together, these results provide some support for the hypothesis that heat tolerance can be limited by insufficient oxygen (Laetz et al., 2024; Pörtner, 2001; Verberk et al., 2013), as boosting tissue oxygen levels via photosynthesis improved heat tolerance, whereas inhibiting photosynthesis reduced heat tolerance.

Small differences in CT_max_ can result in pronounced differences for long-term survival of chronic heat (Verberk et al., 2023), so the light-induced differences reported here are likely ecologically relevant. Future studies into their vulnerability to climate warming would be promising, requiring a slightly different methodology which explicitly takes the temporal dimension into account (Rezende et al., 2014).

### Mechanisms determining heat tolerance in cnidarians

Our results also highlight that mechanisms other than oxygen production could be at play. In *C.* cf. *xamachana*, thermal tolerance increased with increasing light intensity, even in photosynthetically inhibited specimens where metabolic measurements did not indicate oxygen production. Because visual systems and visually guided behaviors are common in cnidarians (Gornik et al., 2021; Picciani et al., 2018), a link between light exposure and various physiological processes including thermal tolerance is plausible. In this light (pun intended), it is interesting that *C.* cf. *xamachana*, the only species with a well-developed visual system and light-dependent behaviors during non-larval ontogenetic stages (Nath et al., 2017), displayed the strongest increase in oxygen consumption when exposed to high light (Fig. 4B), and showed the strongest response to light in heat tolerance when photosynthetically inhibited. Thus, perhaps this species anticipates warmer water when perceiving light, leading to physiological changes that improve heat tolerance and increase metabolic energy demand. Understanding why light influences heat tolerance in our study species will require further study, but it does indicate that light can affect heat tolerance via mechanisms unrelated to oxygen. For example, organisms may use light as a cue to anticipate warmer waters and increase heat tolerance via physiological plasticity in a similar manner as has been observed in response to acclimation to higher temperatures or lower oxygen. This is an important caveat for studies that have documented relationships between light-induced diel fluctuations in oxygen levels and improved heat tolerance in aquatic fauna (Booth et al., 2023).

Light as a cue may also help explain why heat tolerance was unresponsive to light in both photosynthetically inhibited and uninhibited specimens of *M. hargitti* (Fig. 4F). This species is found attached to mangrove roots on the bay's periphery and is therefore more sheltered from direct sunlight by the mangrove trees, whereas *C.* cf. *xamachana* and *B. antilliensis* live in very shallow environments in the middle of the bay, exposed to direct sunlight from dawn to dusk. This species also stands out in other respects. *Myrionema hargitti* is the only species we examined that is completely sessile and therefore unable to move to optimal light/heat conditions once attached to its substrate. It could modulate the light reaching its symbiotic algae by retracting its polyps, but this was not observed in the field, and phototaxis experiments in this species are lacking. Finally, *M. hargitti* lives in colonies of polyps connected by chitinous structures which may have a direct impact on their energy budgeting and heat susceptibility, contrasting the medusa life stage we observed in *C.* cf. *xamachana* and the solitary polyp stage we observed in *B. antillinesis*. Disentangling the specific mechanisms by which light modulates heat tolerance in cnidarian species will require an expansion of the work done here, to examine multiple species from each of these cnidarian clades so that patterns in heat tolerance, metabolism, ecology and anatomy can be identified.

Lastly, photosynthesis provides animals not just with oxygen as a waste product, but also with photosynthates such as glucose. A lack of oxygen and hence energy during experimental ramping trials can be partly compensated via anaerobic metabolism and upregulation of pathways such as the pentose phosphate pathway, which constitutes a major metabolic hub with connections to glycolysis and the TCA cycle (Verberk et al., 2013), and which also helps to explain effects of starvation on heat tolerance (Laetz et al., 2024). However, in our study, consistent differences across specimens in rates of photosynthesis and hence glucose storage prior to experimentation is unlikely because animals were allocated to different light intensities after the inhibition of photosynthesis and just before we started the respiration and thermal tolerance trials.

In conclusion, we show that heat tolerance is modulated by light intensity in two of the three species studied. The reduced heat tolerance following chemical inhibition of photosynthesis indicates a role for tissue oxygen levels and lends support to the hypothesis that oxygen limitation can affect heat tolerance. Because these patterns differ across species, and light perception and mounting anticipatory responses are likely also involved, there is a high probability that mechanisms unrelated to oxygen are also involved in heat tolerance.

## Supplementary Material

10.1242/jexbio.251544_sup1 Supplementary information

## References

[JEB251544C1] Arrhenius, Å., Grönvall, F., Scholze, M., Backhaus, T. and Blanck, H. (2004). Predictability of the mixture toxicity of 12 similarly acting congeneric inhibitors of photosystem II in marine periphyton and epipsammon communities. *Aquat. Toxicol.* 68, 351-367. 10.1016/j.aquatox.2004.04.002 15177952

[JEB251544C2] Booth, J. M., Giomi, F., Daffonchio, D., McQuaid, C. D. and Fusi, M. (2023). Disturbance of primary producer communities disrupts the thermal limits of the associated aquatic fauna. *Sci. Total Environ.* 872, 162135. 10.1016/j.scitotenv.2023.162135 36775146

[JEB251544C3] Breheny, P. and Burchett, W. (2017). Visualization of regression models using visreg. *R J.* 9/2, 56-71. 10.32614/RJ-2017-046

[JEB251544C4] Brijs, J., Jutfelt, F., Clark, T. D., Gräns, A., Ekström, A. and Sandblom, E. (2015). Experimental manipulations of tissue oxygen supply do not affect warming tolerance of European perch. *J. Exp. Biol.* 218, 2448-2454. 10.1242/jeb.121889 26026041

[JEB251544C5] Burgués Palau, L., Senna, G. and Laetz, E. M. J. (2024). Crawl away from the light! Assessing behavioral and physiological photoprotective mechanisms in tropical solar-powered sea slugs exposed to natural light intensities. *Mar. Biol.* 171, 50. 10.1007/s00227-023-04350-w

[JEB251544C6] Christa, G., Gould, S. B., Franken, J., Vleugels, M., Karmeinski, D., Händeler, K., Martin, W. F. and Wägele, H. (2014). Functional kleptoplasty in a limapontiodean genus: phylogeny, food preferences, and photosynthesis in *Costasiella* with a focus on *C. ocellifera* (Gastropoda: Sacoglossa). *J. Molluscan Stud*. 80, 499-507. 10.1093/mollus/eyu026

[JEB251544C7] Clarke, A. and Fraser, K. (2004). Why does metabolism scale with temperature? *Funct. Ecol.* 18, 243-251. 10.1111/j.0269-8463.2004.00841.x

[JEB251544C8] Deutsch, C. A., Tewksbury, J. J., Huey, R. B., Sheldon, K. S., Ghalambor, C. K., Haak, D. C. and Martin, P. R. (2008). Impacts of climate warming on terrestrial ectotherms across latitude. *Proc. Natl. Acad. Sci. USA* 105, 6668-6672. 10.1073/pnas.0709472105 18458348 PMC2373333

[JEB251544C9] Gornik, S. G., Bergheim, B. G., Morel, B., Stamatakis, A., Foulkes, N. S. and Guse, A. (2021). Photoreceptor diversification accompanies the evolution of Anthozoa. *Mol. Biol. Evol.* 38, 1744-1760. 10.1093/molbev/msaa304 33226083 PMC8097283

[JEB251544C10] Laetz, E. M. J., Moris, V. C., Moritz, L., Haubrich, A. N. and Wägele, H. (2017). Photosynthate accumulation in solar-powered sea slugs - starving slugs survive due to accumulated starch reserves. *Front. Zool.* 14, 4. 10.1186/s12983-016-0186-5 28115976 PMC5244517

[JEB251544C11] Laetz, E. M., Kahyaoglu, C., Borgstein, N. M., Merkx, M., van der Meij, S. E. and Verberk, W. C. E. P. (2024). Critical thermal maxima and oxygen uptake in *Elysia viridis*, a sea slug that steals chloroplasts to photosynthesize. *J. Exp. Biol.* 227, 246331. 10.1242/jeb.246331 38629207

[JEB251544C12] Leiva, F. P., Calosi, P. and Verberk, W. C. E. P. (2019). Scaling of thermal tolerance with body mass and genome size in ectotherms: a comparison between water-and air-breathers. *Philos. Trans. R. Soc. B* 374, 20190035. doi:10.1098/rstb.2019.0035 10.1098/rstb.2019.0035 PMC6606457 31203753

[JEB251544C13] McLachlan, R. H., Price, J. T., Solomon, S. L. and Grottoli, A. G. (2020). Thirty years of coral heat-stress experiments: a review of methods. *Coral Reefs* 39, 885-902. 10.1007/s00338-020-01931-9

[JEB251544C14] Nagelkerken, I., Dorenbosch, M., Verberk, W. C. E. P., Cocheret de la Morinière, E. and van der Velde, G. (2000). Day–night shifts of fishes between shallow-water biotopes of a Caribbean bay, with emphasis on the nocturnal feeding of Haemulidae and Lutjanidae. *Mar. Ecol. Prog. Ser.* 194, 55-64. 10.3354/meps194055

[JEB251544C15] Nath, R. D., Bedbrook, C. N., Abrams, M. J., Basinger, T., Bois, J. S., Prober, D. A., Sternberg, P. W., Gradinaru, V. and Goentoro, L. (2017). The jellyfish *Cassiopea* exhibits a sleep-like state. *Curr. Biol.* 27, 2984-2990. 10.1016/j.cub.2017.08.014 28943083 PMC5653286

[JEB251544C16] Picciani, N., Kerlin, J. R., Sierra, N., Swafford, A. J. M., Ramirez, M. D., Roberts, N. G., Cannon, J. T., Daly, M. and Oakley, T. H. (2018). Prolific origination of eyes in Cnidaria with co-option of non-visual opsins. *Curr. Biol.* 28, 2413-2419.e4. 10.1016/j.cub.2018.05.055 30033336

[JEB251544C17] Pinsky, M. L., Eikeset, A. M., McCauley, D. J., Payne, J. L. and Sunday, J. M. (2019). Greater vulnerability to warming of marine versus terrestrial ectotherms. *Nature* 569, 108-111. 10.1038/s41586-019-1132-4 31019302

[JEB251544C18] Pörtner, H. (2001). Climate change and temperature-dependent biogeography: oxygen limitation of thermal tolerance in animals. *Naturwissenschaften* 88, 137-146. 10.1007/s001140100216 11480701

[JEB251544C19] Råberg, S., Nyström, M., Erös, M. and Plantman, P. (2003). Impact of the herbicides 2, 4-D and diuron on the metabolism of the coral *Porites cylindrica*. *Mar. Environ. Res.* 56, 503-514. 10.1016/S0141-1136(03)00039-4 12860435

[JEB251544C20] Rezende, E. L., Castañeda, L. E. and Santos, M. (2014). Tolerance landscapes in thermal ecology. *Funct. Ecol.* 28, 799-809. 10.1111/1365-2435.12268

[JEB251544C21] Seibel, B. A. and Deutsch, C. (2020). Oxygen supply capacity in animals evolves to meet maximum demand at the current oxygen partial pressure regardless of size or temperature. *J. Exp. Biol.* 223, jeb210492. 10.1242/jeb.210492 32376709

[JEB251544C22] Sunday, J. M., Bates, A. E. and Dulvy, N. K. (2012). Thermal tolerance and the global redistribution of animals. *Nat. Clim. Change* 2, 686-690. 10.1038/nclimate1539

[JEB251544C23] Verberk, W. C. E. P., Bilton, D. T., Calosi, P. and Spicer, J. I. (2011). Oxygen supply in aquatic ectotherms: Partial pressure and solubility together explain biodiversity and size patterns. *Ecology* 92, 1565-1572. 10.1890/10-2369.1 21905423

[JEB251544C24] Verberk, W. C. E. P., Sommer, U., Davidson, R. L. and Viant, M. R. (2013). Anaerobic metabolism at thermal extremes: a metabolomic test of the oxygen limitation hypothesis in an aquatic insect. *Integr. Comp. Biol.* 53, 609-619. 10.1093/icb/ict015 23604617 PMC3776598

[JEB251544C25] Verberk, W. C. E. P., Overgaard, J., Ern, R., Bayley, M., Wang, T., Boardman, L. and Terblanche, J. S. (2016). Does oxygen limit thermal tolerance in arthropods? A critical review of current evidence. *Comp. Biochem. Physiol. A. Mol. Integr. Physiol.* 192, 64-78. 10.1016/j.cbpa.2015.10.020 26506130 PMC4717866

[JEB251544C26] Verberk, W. C. E. P., Hoefnagel, K. N., Peralta-Maraver, I., Floury, M. and Rezende, E. L. (2023). Long-term forecast of thermal mortality with climate warming in riverine amphipods. *Glob. Change Biol.* 29, 5033-5043. 10.1111/gcb.16834 37401451

[JEB251544C27] Wang, T., Lefevre, S., Iversen, N. K., Findorf, I., Buchanan, R. and McKenzie, D. J. (2014). Anaemia only causes a small reduction in the upper critical temperature of sea bass: is oxygen delivery the limiting factor for tolerance of acute warming in fishes? *J. Exp. Biol.* 217, 4275-4278. 10.1242/jeb.089920 25394629

[JEB251544C28] Wickham, H. (2016). *ggplot2: Elegant Graphics for Data Analysis*. Springer.

[JEB251544C29] Winterstein, H. (1905). Wärmelähmung und Narkose. *Z. Allg. Physiol.* 5, 323-350.

