# Supplementary material for: Does heat tolerance vary with rates of oxygen production in photosymbiotic cnidarians?
Source: J Exp Biol. 2026 Feb 12;229(3):jeb251544. doi: 10.1242/jeb.251544 (PMC12951609; doi:10.1242/jeb.251544)
Supplement: Supplementary information [file jexbio-229-251544-s1.pdf]

Supplementary Info 1 Detailed methods for measuring oxygen consumption / production

To measure the metabolic rate of each specimen, it was placed in a respiratory chamber (glass jar), containing aerated seawater (with or without monolinuron) and a PSt3 optical sensor (Presens GmbH, Germany). A Fibox 4 Trace (Presens GmbH, Germany) was used to record the oxygen saturation in each respiratory chamber, which was measured every 6 minutes for an hour. Before experimentation, the Fibox was calibrated using 2-point calibration (100% O2 saturated seawater and 0% oxygen: nitrogen gas). Both *B. antillinesis* and *M. hargitti* were measured in 2ml chambers, whereas the larger *C.* cf. *xamachana* was measured in 22ml chambers. Once the specimen was in the chamber, the entire chamber was placed back in the tank where the animal had been housed for 30 minutes, with the lid off to ensure the water in the chamber could mix with the rest of the aerated water in the tank. This provided each specimen some time to recover from the stress of moving to a new environment, and, for the anemones, time to reattach their pedal discs to the chamber wall as a new substrate, without affecting the oxygen saturation in the chamber. After this recovery period, the chambers were closed, ensuring no air bubbles were contained within the chamber and that water could no longer mix. Each chamber was then placed in a larger water bath to maintain a stable temperature of 26°C. Blank chambers were also examined using water from the same tanks that each specimen had been in, to measure the amount of background microbial respiration.

To examine oxygen uptake or release during the respirometry experiments, specimen volumes were first subtracted from the total chamber volume (2ml or 22ml) resulting in the actual amount of water (and hence oxygen) in the chamber (i.e. larger specimens take up more space so less water is therefore contained in these chambers). Measuring the volume of water displaced by *B. antilliensis* and *M. hargitti* was not possible due to their small size, so it was estimated using a density proxy (1.14 g/l) that was empirically derived from very large *B. antilliensis* specimens (pedal disk diameter 3-4cm) found at the same location as the smaller ones used in this study. The initial and final oxygen saturations (%) were

converted to O_2 concentrations in mgO_2 L^{-1} by determining how much oxygen (mg) is found in 1L of seawater at 26°C and 35 PSU using an online calculator: https://water.usgs.gov/water-resources/software/DOTABLES/ - U.S. Geological Survey, 2018, and then calculating the amount of O_2 actually inside the chamber. The final O_2 concentration was then subtracted from the initial, yielding the change in O_2 concentration during the experiment. Then the amount of background microbial respiration was subtracted. This value was then divided by the time the experiment ran resulting in an amount of O_2 that was produced/consumed by each specimen in one hour (mg O_2 produced/consumed h^{-1}).

Journal of Experimental Biology • Supplementary information

Specimens were fixed in 70% ethanol and then weighed to eliminate variation in their mass due to water in their hydrostatic skeletons, which would influence their wet mass. Since rates of oxygen uptake scale allometrically with body mass [1,2], we could not express rates of oxygen uptake on a per gram basis. Instead, we corrected rates of oxygen uptake using a scaling exponent (0.61) that was derived empirically based on the mass of the specimens and the metabolic rates measured in this study (Fig. S1). Therefore, metabolic rates are presented as mg O_2 produced/consumed $g^{-0.61}$ h^{-1} . This accounts for the effect of mass on metabolic rate and ensures that the remaining variation explained in our statistical analyses is due to our treatments rather than variation in mass across our specimens. All jars were sterilized in between uses.

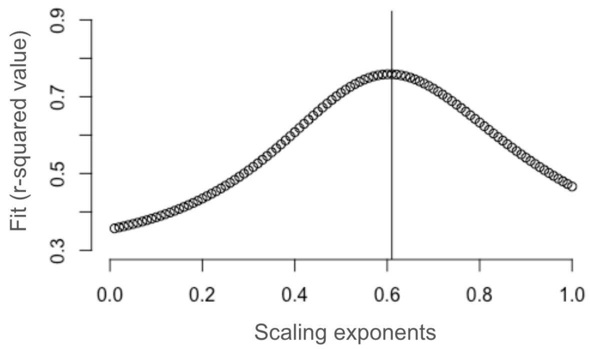

Fig. S1. The mass-specific scaling exponent was determined by calculating r-squared values for all possible scaling exponents (0.0 - 1.0) and selecting the highest r-squared (0.61) as the best fit for the species we examined.

1. White CR, Cassey P, Blackburn TM. 2007 Allometric exponents do not support a universal metabolic allometry. *Ecology* **88**, 315–323. (doi:https://doi.org/10.1890/05-1883)

2. Rubalcaba JG, Verberk WCEP, Hendriks AJ, Saris B, Woods HA. 2020 Oxygen limitation may affect the temperature and size dependence of metabolism in aquatic ectotherms. *Proc. Natl. Acad. Sci.* **117**, 31963–31968.

Table S1. Overview of treatments and number of specimens used. The high-light treatment matches the light intensity measured in the field. The workflow for each specimen was the same (other than the fact that half were exposed to monolinuron). It consisted of collection, transport to the lab, acclimation to lab conditions (~3 hours, half of the specimens were exposed to monolinuron during the last hour and a half of this period), placement in the respiratory chambers, 30 min acclimation to the chambers (with or without monlinuron), removal from chambers and placement in a new jar with 100% saturated seawater for critical thermal experiments, 30 minute acclimation, critical thermal limit experiments, specimen recovery (~1 hour) and lastly fixation for accurate mass measurements.

	high light<br>1600 µmol $m^{-2}s^{-1}$	moderate light<br>700 µmol $m^{-2}s^{-1}$	low light<br>100 µmol $m^{-2}s^{-1}$
Non-monolinuron-treated (photoysnthetic)	n=9 of each species	n=9 of each species	n=9 of each species
monolinuron-treated	n=9 of each species	n=9 of each species	n=9 of each species

Journal of Experimental Biology • Supplementary information

Table S2. Summary statistics from the O_2 production/consumption experiments. Since "Light intensity" had three levels, we log transformed it to account for non-linear effects. The bestfit model allowed interactions between the predictors, "Species", "log10Light" and "Monolinuron".

term	estimate	std.error	t value	p value
(Intercept)	-0.072	0.018	-3.93	**0.00013**
SpeciesCassiopea_xamachana	0.027	0.026	1.028	0.31
SpeciesMyrionema_hargitti	-0.00048	0.026	-0.018	0.99
log10Light	0.028	0.0067	4.13	**6.05E-05**
Monolinuron_y	-0.015	0.026	-0.59	0.56
SpeciesCassiopea_xamachana : log10Light	0.0091	0.0095	0.96	0.34
SpeciesMyrionema_hargitti : log10Light	0.014	0.0096	1.49	0.14
SpeciesCassiopea_xamachana : Monolinuron_y	0.096	0.037	2.61	**0.0098**
SpeciesMyrionema_hargitti : Monolinuron_y	0.049	0.037	1.33	0.18
log10Light : Monolinuron_y	-0.010	0.0095	-1.10	0.27
SpeciesCassiopea_xamachana : log10Light:Monolinuron_y	-0.053	0.013	-3.91	**0.00014**
SpeciesMyrionema_hargitti : log10Light:Monolinuron_y	-0.035	0.014	-2.58	**0.011**

Journal of Experimental Biology • Supplementary information

Table S3. Summary statistics from the Critical Thermal limit experiments. The best fit model included interactions between the predictors "Species", "log10(Light)" and "Monolinuron" as well as an interaction between "CT_type" and "Species" and additive effects from "CTtype".

term	estimate	std.error	t value	p value
(Intercept)	28.74	1.012	28.39	**7.38E-88**
CTtype	3.16	0.13	23.85	**7.05E-72**
SpeciesCassiopea_xamachana	0.30	1.43	0.21	0.83
SpeciesMyrionema_hargitti	5.50	1.43	3.84	**0.00015**
log10Light	1.68	0.37	4.52	**8.83E-06**
Monolinuron_n	-2.43	1.43	-1.70	0.091
CTtype : SpeciesCassiopea_xamachana	-1.87	0.19	-9.99	**1.54E-20**
CTtype : SpeciesMyrionema_hargitti	-1.30	0.19	-6.91	**2.86E-11**
SpeciesCassiopea_xamachana : log10Light	1.47	0.52	2.80	**0.0054**
SpeciesMyrionema_hargitti : log10Light	-1.16	0.52	-2.21	**0.028**
SpeciesCassiopea_xamachana : Monolinuron_n	4.90	2.025	2.42	**0.016**
SpeciesMyrionema_hargitti : Monolinuron_n	3.73	2.03	1.84	0.067
log10Light : Monolinuron_n	1.53	0.52	2.92	**0.0037**
SpeciesCassiopea_xamachana : log10Light:Monolinuron_n	-1.89	0.74	-2.55	**0.011**
SpeciesMyrionema_hargitti : log10Light:Monolinuron_n	-1.48	0.75	-1.99	**0.048**

Journal of Experimental Biology • Supplementary information

Table S4. Raw data showing the decrease in light with increasing depth from the first day of specimen collection. The values observed in the field determined the light intensities used in the metabolic rate and thermal limit experiments. The mean light intensity at 0m depth (the water's surface) was 1986.58 ± 154.72 µmol m^{-2} s^{-1} (mean ± SD), decreasing to 1624.45 ± 207.74 µmol m^{-2} s^{-1} at 2m depth and 1277.62 ± 149.70 µmol m^{-2} s^{-1} at 4m depth.

Depth	Time	Irradiance (µmol m-2 s-1)
0	12:00:15	2224.7628
2	12:03:45	1694.9253
4	12:05:00	1148.6631
0	12:08:15	2003.8582
2	12:11:30	1795.6148
4	12:14:45	1479.4146
0	12:16:15	1992.09
2	12:19:30	1304.8557
4	12:22:45	1115.5563
0	12:26:45	1807.9323
2	12:29:15	1534.6346
4	12:31:30	1290.2412
0	12:33:30	1904.2314
2	12:35:00	1792.2425
4	12:37:00	1354.2314

Journal of Experimental Biology • Supplementary information
